# Application of distribution functions in accurate determination of interdiffusion coefficients

**DOI:** 10.1038/s41598-018-22992-5

**Published:** 2018-03-22

**Authors:** Ming Wei, Lijun Zhang

**Affiliations:** State Key Laboratory of Powder Metallurgy, Central South University, Changsha, Hunan 410083 China

## Abstract

Diffusion couple technique in combination with the Boltzmann-Matano method is the widely used approach to evaluate the interdiffusion coefficients in the target systems. However, the quality of the evaluated interdiffusion coefficients due to the Boltzmann-Matano method strongly depends on the fitting degree of the utilized continuous function to the discrete experimental composition profiles. In this paper, the application of different types of distribution functions is proposed to solve this problem. For the simple *D-c* relations, the normal, pseudo-normal, skew normal, pseudo-skew normal distributions can be employed, while for the complex *D-c* relations, the superposed distributions should be used. Even for the cases with uphill diffusion, the combined superposition of distributions may be chosen. Through validation in several benchmarks and real alloy systems, accurate diffusion coefficients are proved to be successfully obtained by using the distribution functions. It is anticipated that the Boltzmann-Matano method together with the distribution functions may serve as the general solution for determining the accurate interdiffusion coefficients in different materials.

## Introduction

Diffusion plays an important role in a variety of disciplines^1–4^. Accurate diffusion coefficient, as one of the basic physical properties, is the prerequisite for quantitative description and comprehensive understanding of various phase transformation processes^5–7^. Thus, interdiffusion coefficient deserves numerous theoretical and experimental investigations^8–10^.

The experimental measurements^5,10^ are always the major choices for obtaining the interdiffusion coefficients nowadays though there are significant progress in the atomistic simulations of interdiffusion coefficients, including first-principles calculations^11,12^ and molecular dynamics simulations^13,14^. The mostly used experimental technique for interdiffusivity measurement is the semi-infinite single-phase diffusion couple together with some calculation approaches, like the traditional famous Boltzmann-Matano (B-M) method and its variants^15–19^. The composition profiles of the single-phase diffusion couples along the diffusion direction can be measured by i.e., electronic probe micro-analyzer (EPMA) technique, but the experimental data are always discrete. In order to utilize the Boltzmann-Matano method for calculation of the interdiffusion coefficients, the discrete experimental composition-distance (*c-x*) data should be always fitted by a continuous curve firstly^19^, from which the interdiffusion flux and slope of composition curve can be then easily evaluated. Thus, the accuracy of the calculated interdiffusion coefficients using the B-M method depends largely on the fitting degree to the experimental composition data.

**Figure 1:**
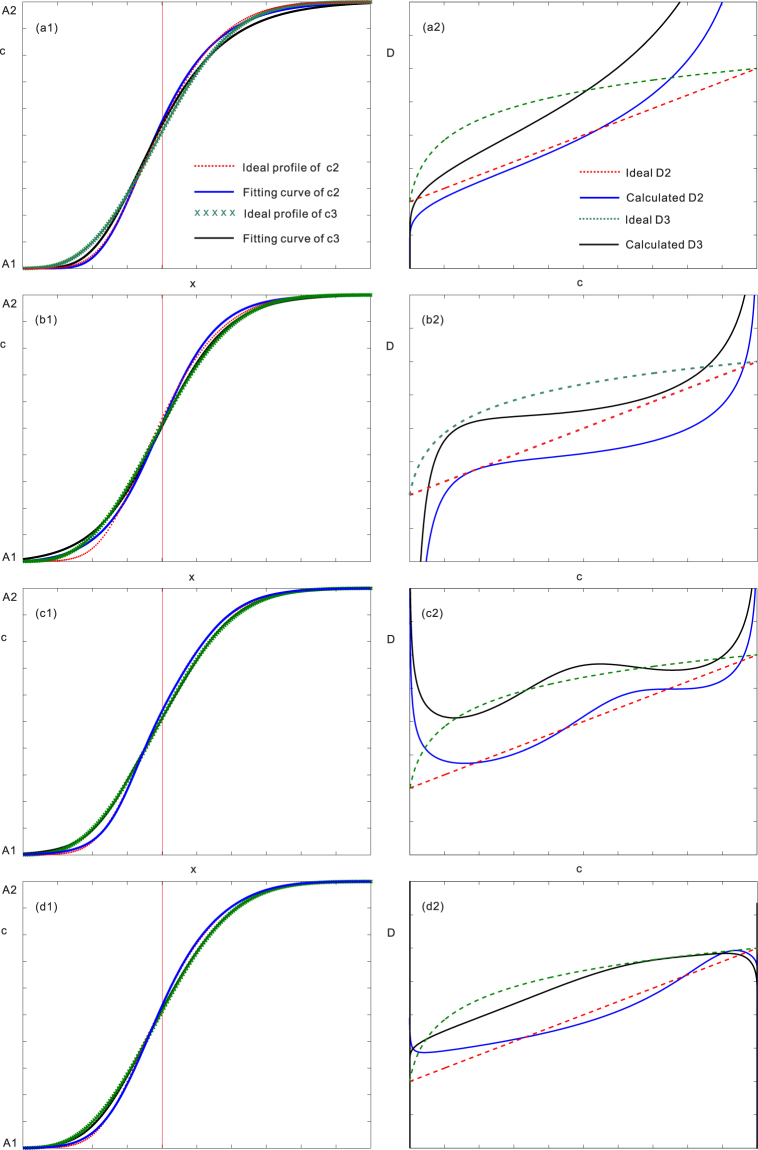
Fitting *c-x* profiles (left plots) and evaluated *D*s (right plots) using different traditional functions, compared with the pre-set concentration profiles of *c*2 and *c*3 and monotonic *D*2 (equation (2)) and *D*3 (equation (3)): (**a**) Nested-exponential function, (**b**) Pseudo-Fermi function, (**c**) superposed-Boltzmann function, (**d**) Superposed-normal distribution function.

**Figure 2:**
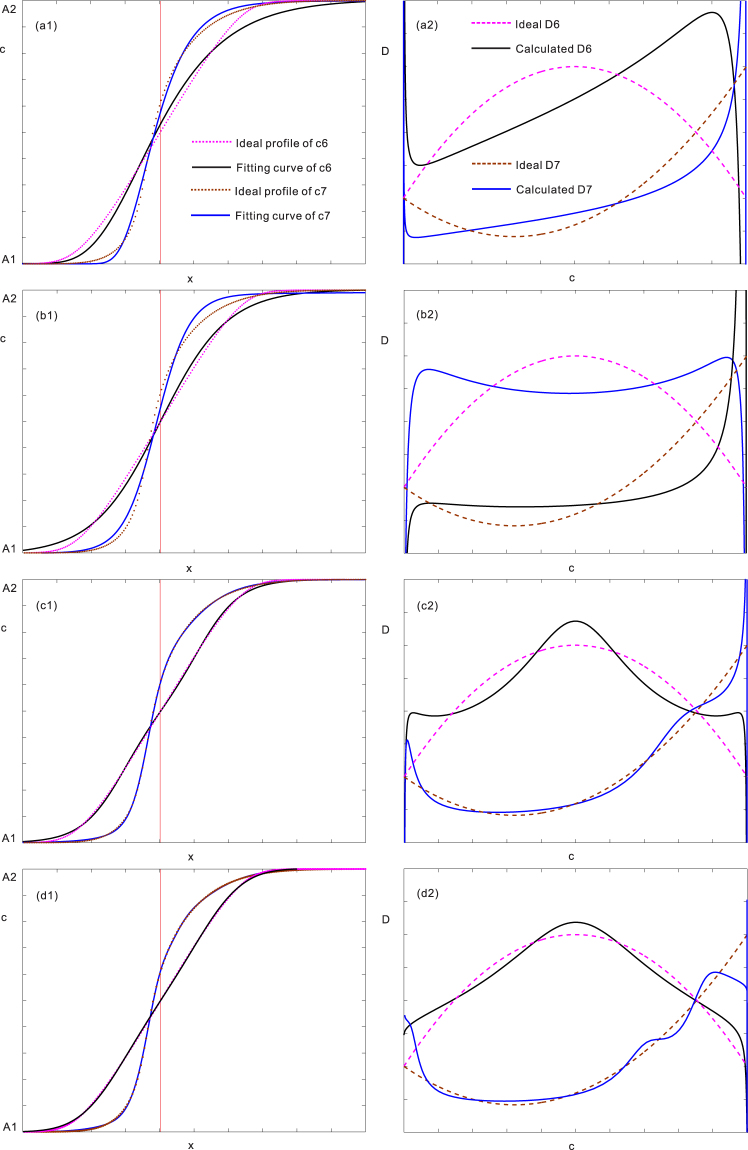
Fitting *c-x* profiles (left plots) and evaluated *D*s (right plots) using different traditional functions, compared with the pre-set concentration profiles of *c*4 and *c*4 and monotonic *D*4 (equation (4)) and *D*5 (equation (5)): (**a**) Nested-exponential function, (**b**) Pseudo-Fermi function, (**c**) superposed-Boltzmann function, (**d**) Superposed-normal distribution function.

Currently, the commonly used fitting functions available in the literature include error function^20,21^, Boltzmann function (logistic function)^22,23^, nested-exponential function^24^, pseudo-Fermi function^25^, and so on. Six types of ideal *D-c* relations (see equations (1–6)) were pre-set in **Methods** section to test the calculation results of these common functions. Although each fitting function can match a majority of the experimental data in different degrees, the calculated interdiffusion coefficients due to different fitting functions may lead to certain differences, as demonstrated in Figs 1 and 2. The left plots in Fig. 1 are the standard *c-x* profiles (i.e., ideal profiles of *c*2 and *c*3) computed using the ideal monotonic *D2* (i.e., equation (2)) and *D3* (i.e., equation (3)) relations in comparison with the fitted *c-x* profiles (i.e., fitting curves of *c*2 and *c*3) using different types of functions. Similarly, the left plots in Fig. 2 are the standard *c-x* profiles (i.e., ideal profiles of *c*4 and *c*5) computed using the ideal parabolic *D4* (i.e., equation (4)) and *D5* (i.e., equation (5)) relations, compared with the fitted *c-x* profiles (i.e., fitting curves of *c*4 and *c*5) using different types of functions. While the right plots in Figs 1 and 2 are the ideal *D-c* relations (i.e., ideal *D*2~*D*5 based on equations (2)~(5)) in comparison with the evaluated *D-c* relations (i.e., calculated *D*2~*D*5) using different fitted functions. As can be seen, the apparent differences between the pre-set *D-c* relations and the calculated ones can be observed.

Consequently, in order to evaluate the accurate interdiffusion coefficients *via* the Boltzmann-Matano method based on the discrete experimental data, the best fitting function should be chosen. In order to achieve this goal, the distribution functions are proposed to fit the discrete experimental composition data in this paper. Though the distribution functions have been widely investigated by many mathematical researchers^26–31^, they focused on the profiles of the probability distribution functions (PDFs). While in this paper, we would rather concern the profiles of the cumulative distribution functions (CDFs). It is well-known that CDFs are non-decreasing, right-continuous and bounded, while the typical plot of CDF behaviors S-shape. Thus we assume that for the normal semi-infinite diffusion couple, its *c-x* profile can be described using a CDF. Moreover, because a variety of CDFs, including the classic normal distribution, skew normal distribution, and more complex ones, like pseudo-normal distribution and pseudo-skew normal distribution, are available for choices, the much more complex *c-x* profile, i.e., with uphill diffusion phenomenon, can be also fitted by the combined distribution functions. In this paper, we will demonstrate the successful application of different distribution functions in accurate determination of composition-dependent interdiffusion coefficients by using benchmarks and real alloys. Moreover, these distribution functions are also proved to be quite qualified for ternary and even multi-component alloys, like high-entropy alloys. It is anticipated that the distribution functions together with the B-M method may serve as a standard solution for evaluation of accurate interdiffusion coefficients.

## Methods

### Pre-set ideal *D-c* relations

In order to test the calculation result of these traditional functions directly, following the idea from Kailasam^32^, 6 types of ideal *D-c* relations are pre-set in this work as benchmarks. *D1* is a constant, *D2* has a linear relation with *c*, *D3* has a logarithm relationship with *c*, *D4* and *D5* have parabolic relations with *c*, while *D6* has normal distribution relations with *c*.

1 $${D}{1}=0.02;$$

2 $${D}{2}={D}{1}+0.004{c}{;}$$

3 $${D}{3}={D}{1}+0.01\times {\rm{l}}{\rm{o}}{\rm{g}}(5.36{c}+1);$$

4 $${D}{4}={D}{1}{-}0.0016{{c}}^{2}+0.016{c}{;}$$

5 $${D}{5}={D}{1}+0.001125{{c}}^{2}-0.00725{c};$$

6 $${D}{6}={D}{1}+0.04\exp (-0.2{({c}-5)}^{2});$$

**Figure 3:**
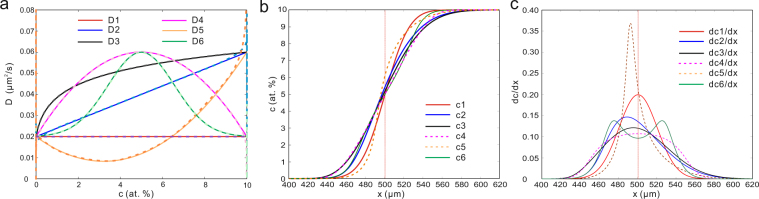
Simulated/calculated different diffusion properties for the case with ideal *D*s. **(a)** Solid lines, 6 types of ideal *D-c* relations; dashed lines, the calculated *D-c* function obtained by applying the B-M analysis to the corresponding CDF in (b). **(b)** *c-x* profiles of the ideal *D*s, corresponding to the profiles of the cumulative distribution functions. **(c)** *dc/dx* profiles of the ideal *D* corresponding to the profiles of the probability distribution functions.

The unit of *D* is ${{\rm{um}}}^{2}/{\rm{s}}$, *c* denotes concentration of solutes in atom percent, the diffusion time is 10000 seconds, the length of *x* is 1000 ${\rm{um}}$. Initially, c equals to 0 where ${\rm{x}}\le 500$ and equals to 1 where ${\rm{x}} > 500$. The steps of time and distance in the diffusion simulation are 1 second and 1 $\,{\rm{um}}$, respectively. Insulation boundary condition and finite difference method are applied in the iteration. The solid lines in Fig. 3a show the profiles of the pre-set *D-c* relations and Fig. 3b shows the simulated *c-x* profiles.

### Traditional fitting functions

For traditional fitting functions, error function and Boltzmann function are often used to describe the symmetric experimental composition profiles. Meanwhile, the nested-exponential function, pseudo-Fermi function, superposed-logistic function and superposed-error function are also used to fit the unsymmetrical experimental data. These traditional functions, except for the superposed form, are presented as follows,

7 $${\rm{E}}{\rm{r}}{\rm{r}}{\rm{o}}{\rm{r}}\,\text{function}:\,{{c}}_{{E}}({x})={p}1\times {\rm{e}}{\rm{r}}{\rm{f}}(-\frac{{x}-{p}2}{{p}3})+{p}4;$$

8 $${\rm{B}}{\rm{o}}{\rm{l}}{\rm{t}}{\rm{z}}{\rm{m}}{\rm{a}}{\rm{n}}{\rm{n}}\,\text{function}:\,{{c}}_{{B}}({x})=\frac{{p}1}{1+\exp (-\frac{{x}-{p}2}{{p}3})}+{p}4;$$

9 $$\text{Nested} \mbox{-} \text{exponential}\,\text{function}:\,{{c}}_{{N}}({x})={\rm{p}}1\times \exp (-\exp ({\rm{p}}2-{\rm{p}}3\cdot {x}))+{\rm{p}}4;$$

10 $$\text{Pseudo} \mbox{-} \text{Fermi}\,\text{function}:\,{{c}}_{{F}}({x})=\frac{{p}1-{p}2\cdot {x}}{1+\exp (-\frac{{x}-{p}3}{{p}4})}+{p}5;$$

Afterwards, nested-exponential function, pseudo-Fermi function, 3 order superposed-Boltzmann function and 3 order superposed-error function are applied to repeat the curves of ideal *D2*, *D3*, *D4* and *D5* by fitting corresponding *c-x* profiles. The calculation results have been already shown in Figs 1 and 2. As can be seen, although each fitting function can match a majority of the experimental data in different degrees, the calculated interdiffusion coefficients due to different fitting functions may lead to certain differences.

Therefore, a series of distribution functions are proposed to accurately determine the interdiffusion coefficients. No matter how complex the *D-c* relation is, the experimental *c-x* profile can be treated as a cumulative distribution function and its slope *dc/dx-x* profile can be treated as a probability distribution function. Moreover, the normal distribution is the numerical solution of the *c-x* profile with constant interdiffusion coefficient, and the term $\lambda =({x}-{{x}}_{{0}})/\sqrt{2{D}{t}}$ corresponds to the variable $({x}-{u})/\sqrt{2}\sigma$ in the normal CDF. Therefore, normal distribution and its derivatives are chosen as the description functions, as demonstrated in the following. It should be noted that in this paper the fitting curves of common functions are denoted as *c(x)* distinguished using different subscripts, while the fitting curves of distribution functions are denoted as *F(x)* or *G(x)*. *F(x)* represents normal CDF and pseudo-normal CDF, while *G(x)* represents skew normal CDF and other complex CDF.

### Normal distribution and simple symmetrical ***D****-c* relations

The general expression for normal distribution is,

11-1 $$F(x)={F}_{1}(x)={\int }^{}{f}_{1}(x)dx=p1+p2\times {\int }^{}exp(-p3\times {(x-p4)}^{2})dx$$

It is the numerical solution for constant interdiffusion coefficient. *n* in ${f}_{n}({\rm{x}})$ means the number of normal distribution functions. For simple symmetrical *D-c* relations, normal distribution could be modified as

11-2 $$F(x)={F}_{p5}(x)={\int }^{}{f}_{p5}(x)dx=p1+p2\times {\int }^{}exp(-p3\times {(x-p4)}^{2\times p5})dx$$

A parameter $p5$ is used to adjust *x* axis. For simple symmetrical convex *D-c* relations, $p5 > 1$, while for simple symmetrical concave parabolic *D-c* relations, $p5 < 1$. When $p5=1$, ${f}_{p5}(x)$ evolves to ${f}_{1}(x)$. ${f}_{p5}(x)\,$and ${f}_{1}({\rm{x}})$ are both treated as the fundamental distribution functions in this work. If equation (11) does not work well, the following equation is recommended,

12 $$F(x)={\int }^{}[{f}_{x}(x)\times {f}_{p5}(x)]dx$$

Here, ${f}_{x}(x)$ is used to modify the kurtosis, and its form may be a exponential term (like normal PDF), quadratic function, etc. Equations (11–2) and (12) is named as the pseudo-normal distribution in this work. Taking symmetrical parabolic *D4* for instance, equation (13) can give the good fitting result. Its specific expression reads as

13 $$F(x)=p1+p2\times \int [exp(-p3\times {(x-p4)}^{2})\times exp(-p6\times {(x-p7)}^{2\times p5}))]dx$$

The number of constant *D* is used to judge the complexity of the distribution functions. One $f({\rm{x}})$ term (exponential term) corresponds to a constant *D*. Thus, one constant *D* is used in equation (11) and two constant *D*s are used in equation (12).

As Fig. 1b shows, the concave D-c relations could be reproduced with the Boltzmann function, therefore, for concave cases, the Boltzmann function might be also a good choice.

### Skew normal distribution and monotonic ***D-c*** relations

The skew normal distribution is used to fit the *c-x* profiles with monotonic *D-c* relations. The expression of skew distribution function^27,28^ G$(x)$ is shown in equation (14), ${f}_{1}(x)$ denotes the normal PDF and controls the kurtosis of the distribution, ${F}_{2}(x)={\int }^{}{f}_{2}(x)dx$ denotes the normal CDF and controls the skewness of the distribution^29^. Two constant *D*s are used in equation (14).

14 $$\begin{array}{rcl}G(x) & = & {\int }^{}g(x)dx={\int }^{}[{f}_{1}(x)\times {F}_{2}(x)]dx\\  & = & p1+p2\times {\int }^{}[exp(-p3\times {(x-p4)}^{2})\times {\int }^{}{\rm{e}}{\rm{x}}{\rm{p}}(-p5\times {(x-p6)}^{2})dx]dx\end{array}$$

### Pseudo-skew normal distribution and simple unsymmetrical *D-c* relations

Based on the work of skew normal distribution, it is easy to tackle unsymmetrical parabolic *D-c* relations by just adding a G$(x)$ term to equation ${f}_{p}({\rm{x}})$. Therefore, the pseudo-skew normal distribution function is proposed here. Taking equation (12) as example, the modified expression reads as follows,

15 $$G(x)={\int }^{}[{f}_{1}(x)\times {F}_{2}(x)\times {f}_{p5}(x)]dx$$

Accordingly, three constant *Ds* are used in equation (15). Similarly, for concave cases, Boltzmann equation could be used.

### Superposed distribution and complex *D-c* relations

Superposed distributions are recommended to tackle complex *D-c* relations especially for the cases in which there are more than one peak on the *dc/dx-x* profiles, like *dc6/dx* in Fig. 3c. Theoretically, all the above distributions might be used for superposition. But in fact, the normal distribution and skew-normal distribution are proposed here, and their superposed expressions are given as,

16 $$G(x)={F}_{2}(x)+{F}_{4}(x)+{F}_{6}(x)+\ldots $$

17 $$G(x)={\int }^{}[{f}_{1}(x)\times {F}_{2}(x)+{f}_{3}(x)\times {F}_{4}(x)+\ldots ]dx$$

However, if the number of constant *D* used in equation (17) is more than four, the superposed-normal distribution function should be the first choice. Superposed distributions are successfully applied in Ni-Pd system. Strictly, the superposed-normal PDF is the same as Gaussian distribution, while the superposed-normal CDF is the same as the superposed-error function.

### Uphill diffusion

Unfortunately, all the above functions above cannot tackle the uphill diffusion phenomenon due to the “swell” in the composition profiles. Equation (18) has been used to describe the *c-x* profile of uphill diffusion^24^, but obviously the values at the terminals of ${{c}}_{{U}}({x})$ cannot be kept constant for the semi-infinite diffusion couples. Therefore, based on skew/normal PDF and CDF, a method which superposes the combined distribution functions (CDF + PDF) is accordingly proposed to solve this problem. The simplest form refers to equation (19),

18 $${{c}}_{{U}}(x)=p9+\frac{p1+p3x+p5{x}^{2}+p7{x}^{3}}{p2+p4x+p6{x}^{2}+p8{x}^{3}}$$

19 $$G(x)={F}_{1}(x)+{f}_{2}(x)$$

20 $$G(x)={F}_{1}(x)+{f}_{2}(x)+{f}_{3}(x)$$

Actually, the idea of the superposing method is to add a normal PDF ${f}_{2}(x)$ which allows the introduction of a swell to a normal CDF ${F}_{1}(x)$. If there are two swells on the composition profile, equation (20) can be used. Furthermore, for some much more complex cases, the normal CDF and PDF can be replaced by skew normal CDF and PDF, respectively. The most complex form for equation (20) is,

21 $$G(x)={G}_{1}(x)+{g}_{2}(x)+{g}_{3}(x)$$

Here, G_1_$(x)$ is skew normal CDF. In general, one should start from the simplest equation (19) for fitting the experimental data and it will be applied in the ternary Cu-Ag-Sn system.

## Results

### Benchmark tests

Different distribution functions shown in equations (11–1), (14), (14), (13), (15) and (16), are applied for ideal *D1* to *D6* profiles respectively. The solid lines in Fig. 3a show the profiles of the pre-set *D-c* relations. Figure 3b shows the *c-x* profiles of the pre-set *D-c* relations, and they correspond to CDFs. Figure 3c shows the *dc/dx-x* profiles of the ideal *D-c* relations and they correspond to PDF*s*. The calculated *D-c* relations due to the chosen distribution functions together with the B-M method are denoted as dashed lines in Fig. 3a. As can be seen, the ideal *D-c* relations can be exactly reproduced by distribution functions in combination with the B-M method.

### Application in real cases

#### Case 1: Constant D

**Figure 4:**
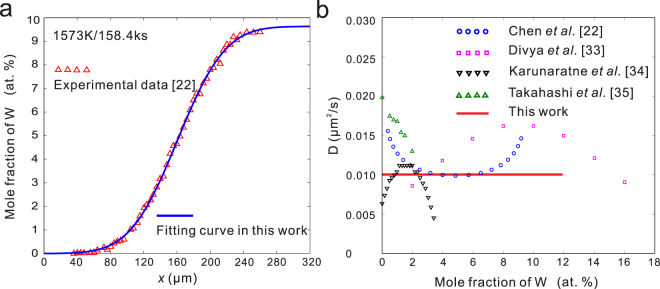
Re-calculated interdiffusion coefficients in Ni-W system^22^ at 1573 K. (**a**) Fitting result of normal distribution function. R^2^ = 0.9992 with R^2^ as the goodness of fit. (**b**) Re-calculated D compared with other researchers’ work^22,33–35^.

Parabolic *D-c* relation in fcc Ni-W system at 1573 K has been obtained in the previous work of Chen *et al*.^22^ from our research group. However, the corresponding *c-x* profile has no characteristic of parabolic *D-c* relations, as shown in Fig. 4a. Therefore, the *c-x* profile is re-fitted with normal distribution function in this work and a constant interdiffusion coefficient is obtained. The results are shown in Fig. 4b. A constant value could reconcile the most results from different researchers^22,33–35^.

#### Case 2: Monotonic D-c relations

The *D-c* relations in most experiments available in the literature are monotonic because of the narrow composition range. Here, the fcc Cu-Sn and Ni-Co systems are taken as the examples of monotonic *D-c* relations.

**Figure 5:**
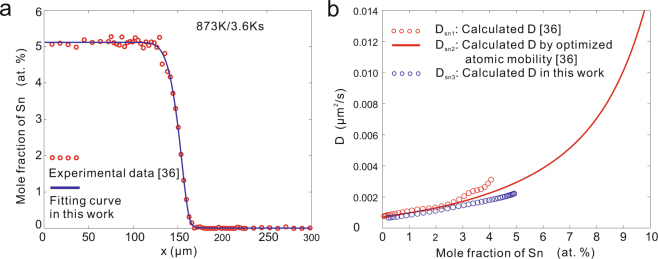
Re-calculated interdiffusion coefficients in Cu-Sn system^36^ at 873 K. **(a)** Fitting *c-x* profile of skew normal distribution function. R^2^ = 0.9986. **(b)** Re-calculated *D* compared with literature data^36^.

For fcc Cu-Sn system, only the previous work by Xu *et al*.^36^ from our research group contains the calculated interdiffusion coefficients and the corresponding *c-x* profile at the same annealing time among all the published papers on fcc Cu-Sn system^36–39^. The smooth interpolation was used in the work of Xu *et al*. while the skew normal distribution function, i.e., equation (14), is applied in this work. The fitting result and the calculated *D* with skew normal distribution function are shown in Fig. 5. As can be seen, the value and the trends of calculated *D* are similar, but larger difference is shown as the content of Sn increases. Moreover, the calculated *D* by Xu *et al*. due to the assessed atomic mobilities in combination with the thermodynamic descriptions is also superimposed in Fig. 5b. As can be seen, the calculated results due to the assessed atomic mobilities approach closely to the present results.

**Figure 6:**
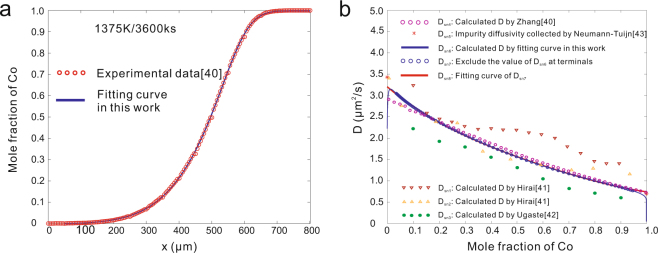
Recalculated interdiffusion coefficients in Ni-Co system^40^ at 1375 K. **(a)** Fitting *c-x* profile of skew normal distribution function. 1 − R^2^ = 7.0757 × 10^−5^. **(b)** Re-calculated *D* in comparison with literature data^41,42,43^.

For Ni-Co system, the delicate experimental results of Zhang and Zhao^40^ are re-analyzed. Zhang and Zhao proposed a forward-simulation method^40^ to make up the deviation of smooth interpolation. The result of the forward-simulation method on Ni-Co system is shown in Fig. 6b. However, this type of *c-x* profile could be tackled simply with skew normal distribution in this work. Figure 6a shows the fitting result of skew normal distribution while Fig. 6b shows the calculated interdiffusion coefficient by different researchers. As can be seen, the results originated from the skew normal distribution and those from the forward-simulation method agree well with each other, but show certain differences from the others^41,42^.

#### Case 3: Parabolic D-c relations

The characteristics of symmetrical and unsymmetrical parabolic *D-c* relations are shown in Fig. 3b. It is difficult to find a real alloy system with strict parabolic *D-c* relation because the convex or concave profile may be complex polynomial. But the pseudo-skew distribution, i.e., equation (15), can be used for approximate parabolic profiles coupling with sectional treatment, taking the *c-x* profile in Nb-W system as an example.

**Figure 7:**
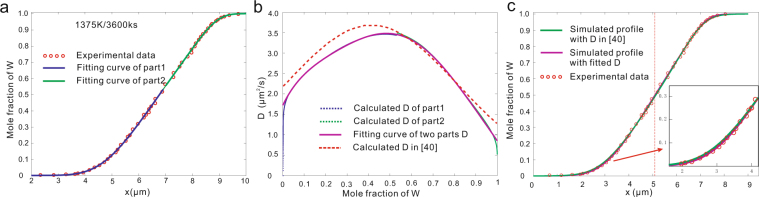
Re-calculated interdiffusion coefficients in Nb-W system^40^ at 1375 K. **(a)** Sectional fitting *c-x* profile with pseudo-skew normal distribution function. 1 − R^2^ = 4.4245 × 10^−4^. **(b)** Re-calculated *D*. **(c)** Simulated c*-x* profile with the re-calculated *D*.

The experimental data also comes from Zhang and Zhao^40^, who also used the forward method. However, in this work, the pseudo-skew distribution function, i.e., equation (15), is directly applied but with sectional treatments. Afterwards two parts of calculated *D*s can be combined and fitted, based on which the composition-profile can be predicted. The two sets of interdiffusion coefficients due to the forward method^40^ and the present pseudo-skew distribution function show slight differences, but both can well repeat the *c-x* profile, as shown in Fig. 7c. Moreover, the extrapolated impurity diffusion coeffecient in the work is more closer to the result of Neumann and Tuijn^43^.

#### Case 4: Complex D-c relations

Generally, the systems which contain complex *D-c* relations typically have a wide solubility range like Ni-Pt^44,45^, Co-Pt^44,46^ and Ni-Pd^47^ systems. The Ni-Pd system^47^ is chosen as an example of complex *D-c* relations in this work.

**Figure 8:**
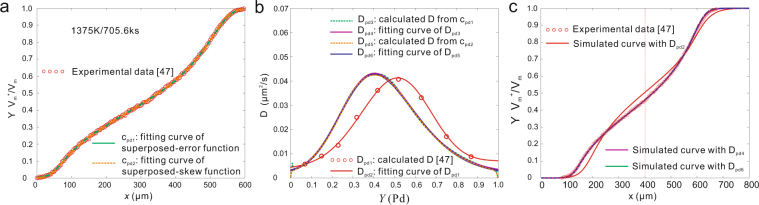
Re-calculated interdiffusion coefficients in Ni-Pd system^47^ at 1375 K. **(a)** Fitting *c-x* profile with superposed-normal distribution function and superposed-skew normal distribution function. 1 − R^2^ = 1.4469 × 10^−4^ and 1.1562 × 10^−4^. **(b)** Calculated *D* with two distribution functions. All the calculated *D* are then fitted by normal PDF. **(c)** Simulated c*-x* profiles with the fitted *D*.

The experimental data are re-fitted by using the superposed-normal distribution function, equation (16), and the superposed-skew normal distribution function, equation (17), respectively, to reveal the advantage of superposed distribution functions in dealing with the concentration profiles of complex D-c relations. The fitting results are presented in Fig. 8a. Afterwards the calculated *D* from the distribution functions together with the calculated *D* by Van Dal *et al.*^47^ are fitted by skew normal PDF, then the diffusion-induced composition profiles is simulated by the fitted *D*.

As can be seen in Fig. 8c, the simulated *c-x* profile in this work shows a very good agreement with the experimental data. In other words, the calculated *D* in this work are more accurate than the literature report. Moreover, the maximum of the interdiffusion coefficient is located at Ni-40Pd at.%, rather than Ni-50 at.% Pd reported by Van Dal *et al*.^47^.

#### Case 5: c-x profiles in ternary system and uphill diffusion

**Figure 9:**
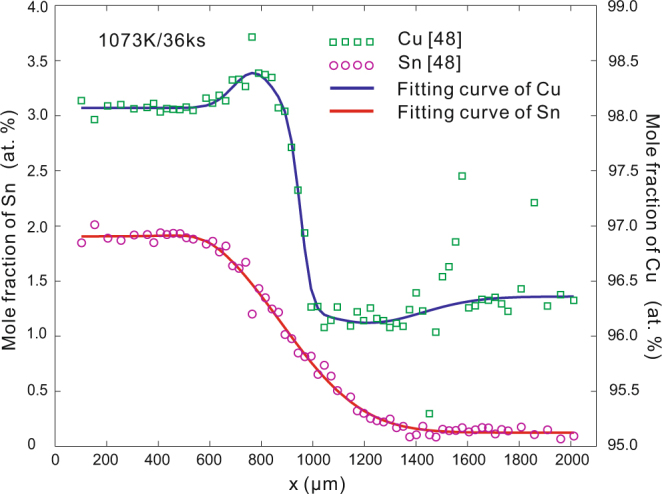
Fitting c-x profiles of Cu and Sn in Cu-Ag-Sn ternary system^48^ at 1073 K, compared with the experimental data^49^. The profiles of Sn and Cu are fitted by skew normal distribution function and combined superposition method respectively, R^2^ = 0.9943 and 0.9323 for Sn and Cu profiles.

The superposed distribution functions can also be applied in ternary and even multi-component systems. The Cu-Ag-Sn system^48^ which contains common *c-x* profile and uphill diffusion profile is taken as an example for ternary systems. The *c-x* profiles of Sn and Cu are described by skew-normal distribution function, equation (14), and the combined superposition method, equation (20), respectively, as shown in Fig. 9. The uphill *c-x* profile of Cu is superposed by one normal CDF and two normal PDFs. The excellent matching results can be seen in Fig. 9.

## Discussion

Although the B-M method has been proposed for decades of years for interdiffusion coefficient calculation, accurate description of the discrete experimental data, as an important step, has not been solved and normalized so far. Disparity between the fitting curve and the experimental data can be easily found in literature reports. It is difficult to evaluate accurate *D-c* relations by using the traditional fitting functions except for some simple cases. Therefore, to explore a standard method which could accurately reveal the real *D-c* relations is imminent. Based on the presently demonstrated successful examples, the distribution functions together with the B-M method is anticipated to serve as a standard solution for accurate interdiffusion coefficient calculation. In the following, we are going to point out some hints on evaluation of accurate interdiffusion coefficients during usage of the proposed distribution functions together with the B-M method.

(*i*) Besides the distribution functions demonstrated in Section *Methods*, there are still some more distribution functions, which can be applied to tackle various different *D-c* relations. Taking the skew normal distribution for instance, the CDF (${F}_{2}(x)$ in equation (14)) could come from normal, Student’s t, Cauchy, Laplace, logistic or the uniform distribution^27^. Of course, the idea of pseudo-normal skew distribution and superposed functions is also applicable in these distribution functions.

(*ii*) It is very important to choose an appropriate distribution function. Although different types of *c*-*x* profiles correspond to different distribution functions, there is a common way to choose an appropriate distribution function. Essentially, the complexity of a distribution function is based on the complexity of a *D-c* relation. Therefore, it is suggested to calculate the general trend of the *D-c* relation using the simple superposed normal distribution function (superposed-error function) first, from which the proper distribution function can be then chosen.

**Figure 10:**
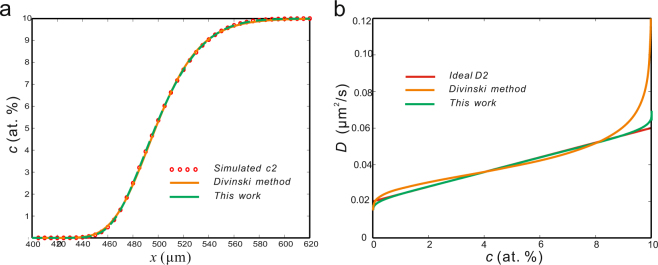
Calculated results for the case ideal *D2* using the method in ref.^49^ and the present method. **(a)** Fitting *c-x* profiles. The goodnesses of fit for the method in ref.^49^ and the present method are 1–5.9836 × 10^−6^; and 1-4.3276 × 10^−8^ respectively. **(b)** Calculated *D* using the two methods, compared with the pre-set *D-c* relation.

**Figure 11:**
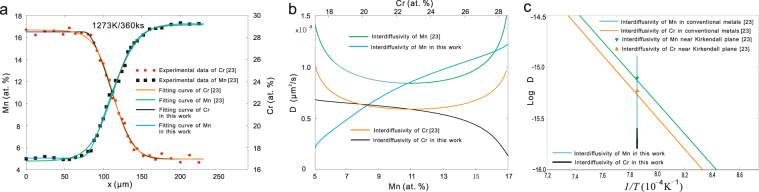
Re-calculated interdiffusion coefficient in fcc quasi-binary CoCrFeMnNi diffusion couple^23^ at 1273 K. **(a)** Fitting *c-x* profiles using Boltzmann function and skew normal distribution function. **(b)** Recalculated *D* in comparison with those by Tsai *et al*.^23^. **(c)** Comparison of interdiffusion coefficients in high entropy alloy and conventional alloys^23^.

Theoretically, the superposed normal distribution function can match any types of *c-x* profiles by adjusting the number of normal CDFs. However, it is not recommended to use the superposed-normal distribution function arbitrarily. As shown in Fig. 1(d), the three-order superposed-normal distribution has a good agreement with the *c-x* profile, but the correspondingly calculated interdiffusion coefficient still has certain deviation from the ideal *D-c* relation. Thus, the superposed normal distribution is only recommended for complex *D-c* relations, such as in the above-demonstrated Ni-Pt system.

(*iii*) Very recently, Kavakbasi *et al*.^49^ proposed a method to calculate the interdiffusion coefficient based on the error function. The idea of their work is to adjust the error function by changing the denominator term while the idea of this work is to adjust the normal CDFs by multiplying terms. The calculated results of the linear *D-c* relation by using the two methods are compared as a benchmark test. As can be seen in Fig. 10, the fitting results from the two methods are both good. The correlation coefficients are both extremely close to one. However, the calculated *D* using the present method has achieved a slightly better result, as shown in Fig. 10b.

(*iv*) When one end-member of the diffusion couple is pure substance, the calculated interdiffusion coefficient at this end should approach to the impurity diffusion coefficient. As displayed in Fig. 5b, the results due to the skew normal distribution at the end of pure element are closer to the impurity diffusion coefficient^43^. One thing to be mentioned here is that the calculated diffusion coefficients at the very close to the edge of the diffusion couple with distribution functions might not tend to a constant sometimes. In this case, one can directly extrapolate from the reliable diffusion coefficients close to the middle part.

(*v*) To determine whether sluggish diffusion exists in high-entropy alloys represents one research hotspot currently. For instance, Tsai *et al*.^23^ designed a quasi-binary diffusion couple to study the diffusion in fcc Co-Cr-Fe-Mn-Ni system. In their work, the B-M method together with the Boltzmann function are applied, and their results do not support any sluggish diffusion phenomenon. After that, Paul^50^ also questioned the diffusion data reported by Tsai *et al*.^23^. In this work, the original experimental data of Cr and Mn from ref.^23^ are re-calculated with the B-M method combined with the skew normal distribution function. Figure 11a shows the fitting results of the experimental data, while Fig. 11b presents the re-calculated interdiffusion coefficients. Different from the two similar *D*-*c* profiles in ref.^23^, the trends of the concentration dependence of diagonal interdiffusion coefficients of Cr and Mn show monotonic increase and monotonic decrease, respectively. In general, most fitting functions could work well for the *c-x* profile in the middle part over the diffusion zone. That is why the interdiffusion coefficients evaluated using the present distribution functions and those using the tradition functions are in the same order. As for the *c-x* profile at the edges of the diffusion zone, the fitting results of distribution functions can gain better agreement with the experimental data than the traditional fitting functions, as shown in Fig. 9(a). Such differences may lead to the difference in the evaluated interdiffusion coefficients (even the variation trend), as demonstrated in Fig. 9(b). Moreover, in this quasi-binary diffusion couple, the diagonal interdiffusion coefficients of Mn at low concentrations are even smaller than those in conventional alloys. While the diagonal interdiffusion coefficients of Cr over the entire concentration range in the experiments are smaller than those in conventional alloys. A conclusion could be drawn here that the sluggish diffusion exists in the fcc Co-Cr-Fe-Mn-Ni high entropy alloys for some elements at specific concentrations.

## Conclusions

In a conclusion, the accurate calculation of interdiffusion coefficients can be achieved by the application of distribution functions together with the B-M method. The normal, pseudo-normal, skew normal, pseudo-skew normal distributions are applicable for simple *D-c* relations, while the superposed distributions are applicable for complex *D-c* relations. Moreover, the combined superposition of distributions is proposed for uphill diffusion curve. The application of distribution functions in benchmark and real alloy systems demonstrates that accurate diffusion coefficients can be successfully evaluated. Therefore, the Boltzmann-Matano method in combination with the distribution functions is proved to be the general solution for accurate determination of diffusion coefficients.
